# Acupoint application for rotavirus diarrhea in infants and children: A protocol for systematic review and meta analysis

**DOI:** 10.1097/MD.0000000000022227

**Published:** 2020-09-18

**Authors:** Shaodan Sun, Xiaojie Lin, Yang Yang, Jingtu Cen, Fei Luo, Xiaogang Chen

**Affiliations:** a Department of Pediatrics, The First Clinical Medical School of Guangzhou University of Chinese Medicine, Guangzhou University of Chinese Medicine; b Department of Medical Education Office, Guangdong, Provincial Hospital of Traditional Chinese Medicine, Guangzhou; c Department of Rehabilitation, Affiliated Jiangmen Traditional Chinese Medicine Hospital of Ji’nan University, Ji’nan University, Jiangmen; d Department of Otolaryngology, Tongde Hospital of Zhejiang Province, Hangzhou; e Department of Pediatrics, The First Affiliated Hospital of Guangzhou University of Traditional Chinese Medicine, Guangzhou, China.

**Keywords:** acupoint application, meta-analysis, protocol, rotavirus diarrhea, systematic review

## Abstract

**Background:**

Diarrheal disease currently claims the lives of approximately 500,000 children each year. Rotaviruses are the pathogens primarily responsible for more severe cases and more than one-third of diarrhea-associated deaths in children under 5 years old globally. At present, commonly used drug therapies for rotavirus diarrhea in Western medicine, such as oral rehydration salts, montmorillonite, probiotics, and nitazoxanide, often cannot achieve satisfactory curative effects. Moreover, infants’ and children's compliance with drugs and injections is often lower than their compliance with acupoint application therapy. A large number of studies have shown that acupoint application can increase the clinical cure rate and shorten the duration of diarrhea. However, there is a lack of systematic reviews on the safety and efficacy of acupoint application in the treatment of rotavirus diarrhea. Therefore, we will conduct a study to evaluate the safety and efficacy of acupoint application for rotavirus diarrhea in infants and children.

**Methods:**

We will search the relevant medical literature using PubMed, EMBASE, Web of Science, Cochrane CENTRAL, China National Knowledge Infrastructure, the Wanfang Database, the Chinese Biomedical Literature Database, and the Chinese Scientific Journal Database from inception to August 2020. Both MeSH and free text terms will be utilized to obtain the maximum numbers of papers. No language restrictions will be applied, and the publication type will be limited to randomized controlled trials. Two teams will independently review and assess the studies for inclusion in the review. RevMan V 5.0 software will be applied for data extraction. The methodological quality of the included studies will be evaluated according to the Cochrane Handbook.

**Results:**

The results of this study will be published in a peer-reviewed journal.

**Conclusion:**

The conclusion of this systematic review will provide evidence regarding whether acupoint application is an effective intervention for infants and children with rotavirus diarrhea.

**INPLASY registration number:**

INPLASY202070123.

## 1 Introduction

Diarrheal disease currently claims the lives of approximately 500,000 children every year. Although a series of enteric pathogens contributes to this phenomenon, the cases and hospitalizations caused by rotaviruses are more serious than those caused by any other pathogen. Globally, the virus is responsible for more than one-third of diarrhea-related deaths among children younger than 5 years of age.^[1]^

Rotavirus-induced diarrhea is considered to be noninflammatory, and there are 3 proposed mechanisms: osmotic diarrhea due to malabsorption (secondary to intestinal cell damage or death or due to decreased epithelial absorption) and secretory diarrhea caused by the effects of NSP4 and the activation of the enteric nervous system.^[2]^ Moreover, the secretion of serotonin (5-HT) mediated by rotavirus infection can activate signaling pathways, leading to diarrhea and vomiting.^[3]^

Key treatment concepts include the following: fluid and electrolyte management. Oral rehydration therapy has been safely and successfully used to prevent and treat dehydration caused by rotavirus in infants and young children.^[4]^ Montmorillonite. Montmorillonite as an adjuvant to rehydration therapy can shorten the duration of diarrhea in children with acute infectious diarrhea by 1 day but does not have a significant impact on hospitalization rates or the need for intravenous therapy.^[5,6]^ Nitazoxanide. Nitazoxanide, a broad-spectrum antiviral drug, has been reported to shorten the duration of diarrhea and hospital stay in children with acute rotavirus diarrhea.^[7]^ However, no clinical trial of nitazoxanide for the treatment of rotavirus diarrhea patients under 12 months has been reported. Probiotics. In clinical practice and a number of studies, probiotics have been suggested to be effective at treating various forms of acute and chronic diarrhea.^[8]^ However, many studies have found that probiotics did not significantly improve outcomes compared to a placebo.^[9,10]^

Acupoint application, based on the theories of traditional Chinese medicine (TCM), is a therapy applying Chinese herbal preparations to the skin at acupuncture points. It is an external treatment. Acupoint application involves the direct application of the medicine to the acupoints, which has an effect without passing through the liver and digestive tract, reducing the first-pass effect of liver metabolism and the influence of the pH of the digestive tract on the blood drug concentration and at the same time avoiding the side effects of the drug on the gastrointestinal tract.^[11,12]^ Application therapy is an important part of TCM, and it is simpler and more practical than internal therapy. Importantly, acupoint application is easily accepted by children.

Although acupoint application for the treatment of diarrhea in infants and children diarrhea is common in China, there is still a lack of systematic reviews on the safety and efficacy of the use of acupoint application for the treatment of rotavirus diarrhea. We therefore performed a meta-analysis of currently available randomized controlled trials (RCTs) to evaluate all the clinical evidence on the effectiveness and safety of acupoint application for rotavirus diarrhea treatment.

## 2 Methods

This protocol has been registered in INPLASY under the number INPLASY202070123. If there are any changes, we will describe them in our full review.

### 2.1 Inclusion criteria for study selection

#### 2.1.1 Types of studies

All RCTs of acupoint application therapy for the treatment of rotavirus diarrhea will be included. No language restrictions will be applied.

#### 2.1.2 Type of participants

The inclusion criteria will be as follows: RCTs; infants and children ≤14 years; diarrhea caused by rotavirus, and rotavirus diarrhea diagnosed based on symptoms and stool tests; duration of diarrhea ≤3 days; and diarrhea defined as 3 or more loose stools in a 24 hours period.

#### 2.1.3 Type of interventions

In accordance with earlier Cochrane reviews, only acupoint application for the treatment of rotavirus diarrhea will be considered in the experimental groups.

#### 2.1.4 Type of comparator (s)/control

The control group will be treated with no intervention, sham acupoint application therapy, or a placebo control.

#### 2.1.5 Type of outcome measures

The primary outcomes will include the duration of diarrhea and the frequency of diarrhea. The secondary outcomes will include the duration of fever, the duration of vomiting, the duration of hospitalization, ORS intake, and adverse events.

### 2.2 Search methods for the identification of studies

#### 2.2.1 Electronic searches

We will search the relevant medical literature using PubMed, EMBASE, Web of Science, Cochrane CENTRAL, China National Knowledge Infrastructure, the Wanfang Database, the Chinese Biomedical Literature Database, and the Chinese Scientific Journal Database from inception to August 2020. Both MeSH and free text terms will be utilized to obtain the maximum number of papers. The following search terms will be used: acupuncture point application, application, Rotavirus, rotavirus infection, rotavirus enteritis, rotavirus diarrhea, rotavirus gastroenteritis. No language restrictions will be applied, and the publication type will be limited to RCTs.

### 2.3 Data collection and analysis

#### 2.3.1 Selection of studies

**Figure 1:**
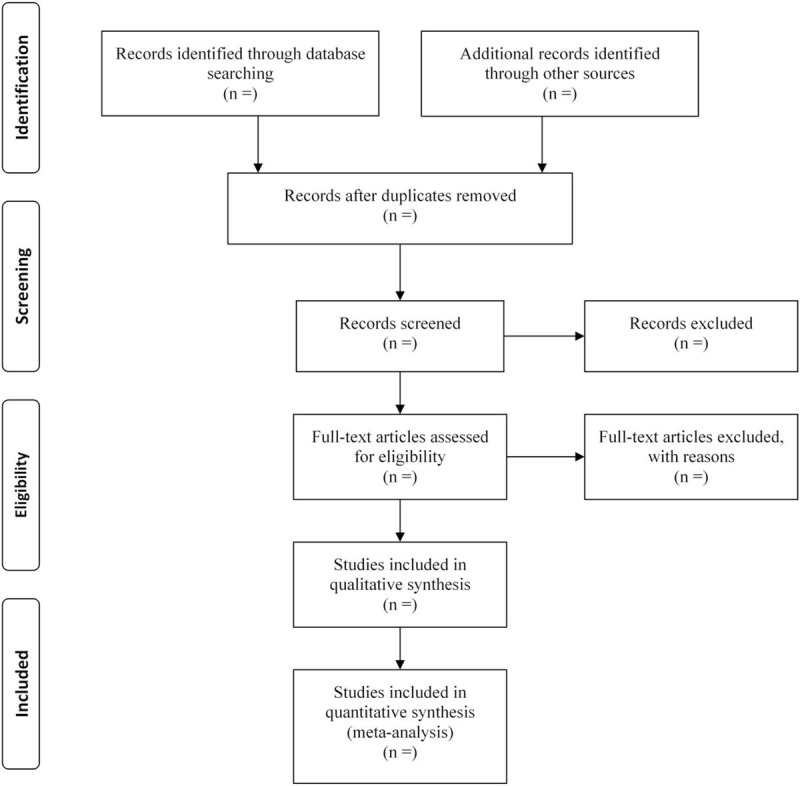
PRISMA flow chart.

Two teams (SS and YY; JC and XL) will independently review and assess the studies to determine which should be included in the review. Discrepancies will be resolved by discussion with XC. If necessary, the original corresponding author can be contacted by email or phone to obtain unclear but very important information. Endnote X9 software will be used to manage the trials that have been identified and remove duplicates. The details of the selection procedure are shown in a PRISMA flowchart (Fig. 1).

#### 2.3.2 Data extraction and management

Data will be extracted by 2 reviewers (SS and XL). The data extracted will include the author, year, region, sample size, age (months and years), intervention (acupoint alone), control intervention, adverse events, and outcomes.

#### 2.3.3 Assessment of risk of bias in included studies

Two independent authors will assess the risk of bias in the included studies using a modification of the assessment in the Cochrane Handbook for Systematic Reviews of Interventions, as follows:

1. selection bias: random sequence generation and allocation concealment;
2. performance bias and detection bias: blinding;
3. attrition bias: incomplete outcome data;
4. reporting bias: selective reporting; and
5. other bias: premature completion.

Each item will be assessed as “low risk,” “unclear risk,” or “high risk.” Any disagreements will be discussed and determined by an arbiter.

#### 2.3.4 Unit of analysis issues

If there are cross-over trials, we will only use the first-phase data. The data of the arm that meets our inclusion criteria will be analyzed when there are more than 2 arms in 1 study. If there are observations at multiple time points, the short-term effect (at the end of treatment) and long-term effect (at the end of follow-up) will be extracted for analysis.

#### 2.3.5 Dealing with missing data

When experimental data are inadequate or missing, we will make an effort to obtain sufficient and comprehensive data from the studies by sending emails or calling the original author. If it is not possible to obtain the data, the incomplete data will be discarded.

#### 2.3.6 Assessment of heterogeneity

To assess heterogeneity, the I^2^ statistic will be calculated. Significant heterogeneity will be determined by I^2^ values greater than 50%. When heterogeneity is significant, a random effects model will be used, and we will explore the heterogeneity using subgroup and sensitivity analyses.

#### 2.3.7 Assessment of reporting biases

Funnel plots will be used to assess the reporting bias when a sufficient number of included RCTs (>10) is available.

#### 2.3.8 Data synthesis

The analysis will be performed using the Cochrane Handbook of Systematic Reviews of Interventions and Review Manager 5.0 (Cochrane Collaboration, Oxford, UK). The dichotomous data will be assessed with the relative risk with the 95% confidence interval (CI), and continuous outcomes will be analyzed using the standard mean difference with the 95% CI. Publication bias will be assessed by funnel plots with Stata software (version 14.0, StataCorp, College Station, TX).

#### 2.3.9 Subgroup analysis

Subgroup analysis will be performed based on age, residence, acupoint, herbs, controls, and outcome measurements to explore the potential causes of heterogeneity.

#### 2.3.10 Sensitivity analysis

A sensitivity analysis will be performed to determine the quality and robustness of the results according to methodological quality, sample size, and analysis issues.

#### 2.3.11 Grading the quality of evidence

According to the Grading of Recommendations Assessment, Development and Evaluation, the evaluation of the quality of the evidence of the main result indicators will be categorized into 4 levels: high, medium, low, and very low.

#### 2.3.12 Ethics and dissemination

Ethics approval and informed consent are not required because this review is not a clinical study and personal information is not involved.

## 3 Discussion

Disease caused by rotavirus is clinically indistinguishable from diarrheal diseases caused by other gastrointestinal pathogens (such as norovirus, enterovirus 40 and 41, astrovirus, *Escherichia coli*, and Salmonella, etc.), but rotavirus infection is often more serious than infections by other agents.^[13]^

Rotavirus vaccine rollout has decreased the global proportion of hospitalized diarrhea cases attributed to rotavirus infection among children younger than 5 years from 38.0% to 23.0%.^[14]^ However, present rotavirus vaccines have many weaknesses; for example, they are less effective precisely where they are needed most. Rotavirus vaccines are highly effective at preventing severe rotavirus disease in developed countries; however, preliminary efficacy studies in Africa and Asia have found that rotavirus vaccines are less effective at preventing severe rotavirus diseases than they are in developed countries, with large regional differences.^[15]^ The investigation of rotaviruses still offers insights into host–enterovirus interactions, and we should continue to investigate this virus.^[13]^

The acupoints most commonly used in TCM to treat diarrhea are located on the abdomen. For example, one of the most commonly used acupuncture points is Shenque (CV8). Because its anatomical site is located in the center of the abdomen, the adjacent organs (such as stomach, liver, gallbladder, pancreas, and intestines) belong to the digestive system. The Shenque acupoint (CV8) has an important role in the treatment of digestive system diseases.^[16]^ Shenque acupoint application of herbal mixtures can improve bowel movement.^[17,18]^

In acupoint application intended to treat infant and child diarrhea, warm-natured Chinese herbs are generally used, such as *Cortex magnoliae officinalis*, *Cortex cinnamomi*, *Radix aconiti coreani*, *Pericarpium Zanthoxyli*, and *Fructus evodiae*. In the 4 properties theory of TCM, herbs of hot or warm nature usually warm up the interior, dispel cold, and support Yang and are therefore used to treat cold syndromes.^[19]^

Acupoint application therapy has been used for more than 1000 years.^[20]^ As it is a physical therapy with few side effects, acupoint application is especially suitable in infants and children. The findings of this systematic review will provide evidence of the use of acupoint application as an adjuvant therapy for the treatment of rotavirus diarrhea in infants and children.

## Author contributions

**Conceptualization:** Xiaogang Chen.

**Data curation:** Yang Yang, Jingtu Cen.

**Formal analysis:** Xiaojie Lin.

**Funding acquisition:** Fei Luo, Xiaogang Chen.

**Investigation:** Fei Luo.

**Methodology:** Shaodan Sun, Yang Yang.

**Software:** Jingtu Cen, Xiaojie Lin.

**Writing – original draft:** Shaodan Sun.

**Writing – review & editing:** Xiaogang Chen, Fei Luo.

## Abbreviations

Abbreviations: CI = confidence interval, ORS = oral rehydration salts, RCTs = randomized controlled trials, TCM = traditional Chinese medicine.

## References

[1] Parker EPK Grassly NC. Enhancing Rotavirus vaccination: a microbial fix? Cell Host Microbe 2018;24:195 6. 30092196 10.1016/j.chom.2018.07.017

[2] Ball JM Tian P Zeng CQ. Age-dependent diarrhea induced by a rotaviral nonstructural glycoprotein. Science 1996;272:101 4. 8600515 10.1126/science.272.5258.101

[3] Hagbom M Istrate C Engblom D. Rotavirus stimulates release of serotonin (5-HT) from human enterochromaffin cells and activates brain structures involved in nausea and vomiting. PLoS Pathog 2011;7:e1002115. 21779163 10.1371/journal.ppat.1002115 PMC3136449

[4] Kotloff KL Nataro JP Blackwelder WC. Burden and aetiology of diarrhoeal disease in infants and young children in developing countries (the Global Enteric Multicenter Study, GEMS): a prospective, case-control study. Lancet 2013;382:209 22. 23680352 10.1016/S0140-6736(13)60844-2

[5] Campbell J. Smectite for acute infectious diarrhoea in children: a Cochrane review summary. Int J Nurs Stud 1036;2020:45. 10.1016/j.ijnurstu.2020.103645 32564885

[6] Pérez-Gaxiola G Cuello-García CA Florez ID. Smectite for acute infectious diarrhoea in children. Cochrane Database Syst Rev 2018;4:Cd011526. 29693719 10.1002/14651858.CD011526.pub2 PMC6494641

[7] Mahapatro S Mahilary N Satapathy AK. Nitazoxanide in acute Rotavirus diarrhea: a randomized control trial from a developing country. J Trop Med 2017;2017:7942515. 28331496 10.1155/2017/7942515 PMC5346365

[8] Chen SY Tsai CN Lee YS. Intestinal microbiome in children with severe and complicated acute viral gastroenteritis. Sci Rep 2017;7:46130. 28397879 10.1038/srep46130 PMC5387401

[9] Freedman SB Williamson-Urquhart S Farion KJ. Multicenter trial of a combination probiotic for children with gastroenteritis. N Engl J Med 2018;379:2015 26. 30462939 10.1056/NEJMoa1802597

[10] Schnadower D Tarr PI Casper TC. Lactobacillus rhamnosus GG versus placebo for acute gastroenteritis in children. N Engl J Med 2018;379:2002 14. 30462938 10.1056/NEJMoa1802598 PMC6358014

[11] Chen X Xu Z. Chen Xl, Xu Zh. Forty Cases of Chronic Non·Atrophic Gastritis of Qi Deficiency of Spleen and Stomach Treated with TCM PlasterTherapy on Shenque. Henan Traditional Chinese Med 2016;36:1783–4.

[12] Yuan X Yao M Fuchun W. Analysis of selection of points and medication rule in treating infantile diarrhea with acupoint application in modern literature. Jilin J Chin Med 2017;37:1189 92.

[13] Crawford SE Ramani S Tate JE. Rotavirus infection. Nat Rev Dis Primers 2017;3:17083. 29119972 10.1038/nrdp.2017.83 PMC5858916

[14] Aliabadi N Antoni S Mwenda JM. Global impact of rotavirus vaccine introduction on rotavirus hospitalisations among children under 5 years of age, 2008-16: findings from the Global Rotavirus Surveillance Network. Lancet Glob Health 2019;7:e893 903. 31200889 10.1016/S2214-109X(19)30207-4 PMC7336990

[15] Hallowell BD Tate J Parashar U. An overview of rotavirus vaccination programs in developing countries. Expert Rev Vaccines 2020;19:529 37. 32543239 10.1080/14760584.2020.1775079 PMC9234970

[16] Cao LX Chen ZQ Jiang Z. Rapid rehabilitation technique with integrated traditional Chinese and Western medicine promotes postoperative gastrointestinal function recovery. World J Gastroenterol 2020;26:3271 82. 32684741 10.3748/wjg.v26.i23.3271 PMC7336322

[17] Li Y Dong Y Jin J. Therapeutic effect of Shenque point on digestive system diseases. J Changchun Univ Traditional Chin Med 2007;23:86.

[18] Wang H Ji L. Location of Shenque point in rats. Zhen Ci Yan Jiu 2007;32:312.

[19] Liu YQ Cheng MC Wang LX. Functional analysis of cultured neural cells for evaluating cold/cool- and hot/warm-natured Chinese herbs. Am J Chin Med 2008;36:771 81. 18711773 10.1142/S0192415X08006223

[20] Gao S. A Complete Collection of Umbilical Therapy of Traditional Chinese Medicine. Jinan: Jinan Press; 1992.

